# Robust contactless fingerprint authentication using dolphin optimization and SVM hybridization

**DOI:** 10.3389/fdata.2025.1641714

**Published:** 2025-12-05

**Authors:** Jenisha Rachel, Ezhilmaran Devarasan

**Affiliations:** Department of Mathematics, School of Advanced Sciences, Vellore Institute of Technology, Vellore, Tamilnadu, India

**Keywords:** contactless fingerprint, feature extraction, HOG algorithm, machine learning, dolphin optimization algorithm, clustering

## Abstract

The field of contactless fingerprint (CLFP) recognition is rapidly evolving, driven by its potential to offer enhanced hygiene and user convenience over traditional touch-based systems without compromising security. This study introduces a contactless fingerprint recognition system using the Dolphin Optimization Algorithm (DOA), a nature-inspired technique suited for complex optimization tasks. The Histogram of Oriented Gradients (HOG) method is applied to reduce image features, with DOA optimizing the feature selection process. To boost prediction accuracy, we fused the DOA with a Support Vector Machine (SVM) classifier, creating a hybrid (DOA-SVM) that leverages the global search prowess of DOA alongside the reliable classification strength of SVM. Additionally, two more hybrid models are proposed: one combining Fuzzy C-Means (FCM) with DOA-SVM, and another combining Neutrosophic C-Means (NCM) with DOA-SVM. Experimental validation on 504 contactless fingerprint images from the Hong Kong Polytechnic University dataset demonstrates a clear performance progression: DOA (91.00%), DOA-SVM (94.07%), FCM-DOA-SVM (96.03%), and NCM-DOA-SVM (98.00%). The NCM-DOA-SVM approach achieves superior accuracy through effective uncertainty handling via neutrosophic logic while maintaining competitive processing efficiency. Comparative analysis with other bio-inspired methods shows our approach achieves higher accuracy with reduced computational requirements. These results highlight the effectiveness of combining bio-inspired optimization with traditional classifiers and advanced clustering for biometric recognition.

## 1 Introduction

Biometric authentication systems have gained vital importance in security applications due to their ability to verify individuals based on distinct physical or behavioral traits. Among the various biometric modalities, fingerprint (*FP*) recognition remains one of the most extensively used methods due to its uniqueness, permanence, and user acceptance. Conventional systems, however, require physical contact with a sensor, raising concerns about hygiene, latent *FP* security, and potential user resistance (Maltoni et al., 2009; Vibert et al., 2023).

The identification of CLFP has also been proposed as a possible substitute, enabling the recognition of *FP* without physical touch with a sensor (Grosz et al., 2021). This method eliminates the limitations of contact-based systems while aiming to deliver comparable authentication performance. Development and large-scale applications of CLFP recognition technologies have been motivated by the widespread availability of high-quality digital cameras in smartphones and other handheld devices. Despite all these developments, CLFP still face serious challenges that affect performance and dependability.

Even with the benefits that CLFP systems confer, there are many challenges for the actual practical use of such systems, which can be long-lasting. The performance can be severely compromised by degradation of image quality, attributable to factors like variations in illumination levels, capture distance to the finger, angular distortions and focus irregularity. Meanwhile, artifacts introduced by capturing the finger image contactless give rise to non-linear geometric distortions due to varying finger pressure and orientation, which complicates feature extraction compared to contact-based systems. Traditional machine learning classifiers often struggle with the inherent variability in CLFP images, while conventional parameter optimization methods are computationally expensive and frequently yield sub-optimal results. Table 1 provides the summary of recent CLFP research studies.

**Table 1:** Summary of recent CLFP studies: task, approach, novelty, dataset.

**References**	**Main task**	**Novelty/focus**	**Dataset used**
(Yin et al., 2020)	Matching	Perspective distortion	2 datasets
(Tan and Kumar, 2020)	Minutiae/Matching	Pose-invariant matching	Public, custom
(Zhang et al., 2021)	Minutiae Extraction	Joint location/direction	3 datasets
(Grosz et al., 2021)	Matching	Mobile, cross-database	3 datasets
(Attrish et al., 2021)	Matching	Real-time, Jetson Nano	IITI-CFD
(Shi et al., 2022)	Matching	Posture/rotation robustness	PolyU, FVC2004
(Dong and Kumar, 2023)	Matching	Cross-modality, privacy	Custom
(Mohamed-Abdul-Cader et al., 2023)	Matching	Multi-finger, ridge orientation	PolyU, others
(Kaplesh et al., 2024)	Classification	ViT for CLFP	ISPFDv1/v2, UNFIT
(Cui et al., 2024)	Matching	3D minutiae, pose	Custom
(Ruzicka et al., 2025)	Segmentation	Fingertip segmentation, FPN	Custom

Most works focus on either deep learning or optimization techniques, with few studies integrating bio-inspired optimization algorithms with advanced clustering methods for CLFP recognition (Yin et al., 2021; Chowdhury and Imtiaz, 2022; Sreehari and Anzar, 2025). Current research lacks systematic approaches to address indeterminacy in CLFP images using neutrosophic or similar fuzzy clustering methodologies. Limited attention has been given to optimizing feature selection through nature-inspired algorithms such as the DOA, particularly when combined with SVM or clustering techniques (Chowdhury and Imtiaz, 2022; Sreehari and Anzar, 2025). While many studies utilize large public datasets; few report results on smaller, challenging datasets (e.g., 504 images) with detailed computational efficiency analysis (Attrish et al., 2021). Despite significant advances in the field, persistent challenges remain in handling pose variation, achieving sensor interoperability, and developing robust liveness detection mechanisms for CLFP systems.

This research strategically selects the DOA as the core optimization technique based on several compelling advantages over contemporary bio-inspired algorithms. Unlike Particle Swarm Optimization (PSO) which may suffer from premature convergence, or Genetic Algorithms (GA) with high computational complexity, DOA demonstrates superior global search capabilities through its unique echolocation-inspired mechanism. DOA's adaptive search behavior, inspired by dolphin hunting strategies, provides better exploration-exploitation balance compared to Whale Optimization Algorithm (WOA), Ant Colony Optimization (ACO), or Gray Wolf Optimizer (GWO), particularly crucial for complex parameter optimization in biometric systems. The selection of Histogram of Oriented Gradients (HOG) for feature extraction is motivated by its robustness to illumination changes and geometric variations—critical factors in CLFP systems. Overall, HOG has advantages over traditional features like Local Binary Patterns (LBP) or Scale-Invariant Feature Transform (SIFT) for capturing important texture and edge information for fingerprint recognition while also improving computational efficiency compared to deep learning methods. SVM is chosen as the base classifier due to its superior performance in high-dimensional feature spaces and strong theoretical foundation. SVM's ability to handle non-linearly separable data through kernel functions makes it ideal for complex biometric classification tasks compared to simpler classifiers like K-Nearest Neighbors (KNN) or Decision Trees.

Despite considerable progress in CLFP recognition, three significant research gaps remain unaddressed. First, there is limited exploration of bio-inspired optimization algorithms like DOA for simultaneous feature selection and parameter optimization in CLFP systems. Second, current approaches rarely address the inherent indeterminacy and noise in CLFP images using advanced mathematical frameworks like neutrosophic logic. Third, most studies focus on large datasets with insufficient analysis of computational efficiency, which is crucial for real-world deployment.

To address the identified research gaps, this paper introduces and rigorously evaluates a series of novel hybrid models for CLFP recognition. Our key contributions are:

- We propose a standalone DOA-based classifier, establishing a baseline using this unexplored optimizer for CLFP.
- We develop a DOA-SVM hybrid, effectively combining DOA's global search capabilities with SVM's discriminative power for enhanced classification.
- We enhance this hybrid further by integrating Fuzzy C-Means (FCM) clustering, creating the FCM-DOA-SVM model to improve feature representation through soft clustering.
- As our core innovation, we introduce the NCM-DOA-SVM hybrid. This model leverages Neutrosophic C-Means (NCM) clustering to directly quantify and manage the uncertainty inherent in CLFP images, thereby increasing robustness in challenging conditions.

We empirically validated all four approaches on a dataset of 504 CLFP images from the PolyU database. Our evaluation provides a comprehensive benchmark, reporting on both accuracy and computational efficiency to offer valuable insights for practical, resource-conscious applications.

The outline of the paper is as follows: Section 2 presents a literature on related work related to CLFP recognition, optimization algorithms, and clustering methods. Section 3 describes the proposed methodology. Section 4 presents the experimental setup, the findings and the thorough discussion. Finally, Section 5 concludes the paper and suggests future work.

## 2 Related work

CLFP recognition has gained significant attention in recent years as an alternative to traditional contact-based systems. (Jawade et al., 2022) proposed one of the early CLFP identification systems using level zero features, demonstrating the feasibility of this approach. (Labati et al., 2014) provided a comprehensive survey of 2D and 3D touchless *FP* technologies, highlighting the challenges and opportunities in this domain. More recently, investigated the performance and standards for CLFP capture, emphasizing the need for robust algorithms to address the inherent variability in contact-less acquisition. (Priesnitz et al., 2021) conducted an in-depth review of touchless 2D *FP* recognition, surveying recent approaches and their limitations. Despite such advancements, CLFP recognition remains hampered by image quality, feature extraction, and classification performance-related issues. Traditional approaches are generally less robust against variability in CLFP images and hence necessitate the use of more robust optimization and classification methods. Bio-inspired optimization algorithms have demonstrated exceptional effectiveness in the solution of complex optimization problems in various disciplines. The DOA, proposed by (Kaveh and Farhoudi, 2013), simulates the dolphin's echolocation capability to detect optimal solutions within a given range of search. The algorithm was utilized effectively in various optimization problems, including feature selection and parameter optimization in classification problems. Other bio-inspired approaches have also been explored for parameter optimization in machine learning (*ML*) systems. (Huang and Wang, 2006) proposed a genetic algorithm-based approach for feature selection and parameter optimization in SVM and demonstrated improved classification performance. Similarly, (Lin et al., 2008) used particle swarm optimization for optimizing SVM parameters and feature selection. The above studies reflect the popularity of utilizing bio-inspired optimization algorithms for the performance improvement of *ML* classifiers. Their application in CLFP recognition, however, particularly in the presence of advanced clustering techniques, is relatively new.

SVM first proposed by (Cortes and Vapnik, 1995), has been widely used in biometric recognition systems due to their rigorous theoretical foundation and better performance in high-dimensional spaces. SVM finds the best hyperplane with the largest class separability and hence is well suited to biometric classifying tasks. SVM performance is highly dependent on parameter optimization, i.e., the regularization parameter C and kernel parameters (such as gamma in the case of the RBF kernel). Traditional parameter optimization methods like grid search and random search are generally computationally demanding and may not always give the best result.

The FCM approach, introduced by (Bezdek et al., 1984), is an extension of traditional clustering methods in the aspect that it allows data points to be a member of multiple clusters to varying degrees. This approach is particularly useful for biometric data when the boundaries of features are fuzzy. The FCM approach has been applied to numerous biometric recognition systems, including *FP*, face, and iris recognition. Its ability to accommodate uncertainty in feature representation makes it an attractive tool for enhancing the effectiveness of biometric classification systems.

NCM is based on neutrosophic set theory introduced by (Smarandache, 2003) and implemented as a clustering algorithm by (Guo and Sengur, 2015), extends fuzzy clustering by incorporating the dimension of indeterminacy. In NCM, each data point is described by three membership functions: truth (belonging to a cluster), indeterminacy (uncertainty), and falsity (non-belonging to a cluster). This approach is particularly beneficial for CLFP images, where quality can be extremely inconsistent and certain regions can contain indeterminate information via blur, shadow, or other artifacts.

Hybrid approaches combining optimization algorithms, *ML* classifiers, and clustering techniques have shown promising results in various biometric recognition tasks. These approaches leverage the strengths of individual methods to address the challenges in biometric recognition, particularly in contact-less scenarios. However, despite the potential of hybrid approaches, there is limited research on combining bio-inspired optimization algorithms like DOA with advanced clustering techniques like FCM and NCM for CLFP recognition. This research gap motivates our proposed frame-work, which aims to improve the accuracy and robustness of CLFP recognition through novel hybrid approaches. Table 2 provides a recent study on the methods analyzed on PolyU CLFP Dataset.

**Table 2:** Summary of recent CLFP studies: task, approach, novelty, dataset.

**References**	**Main task**	**Key techniques**	**Method**
(Peddi et al., 2025)	Minutiae localization, matching	Grouped multi-scale graph-involution, end-to-end learning	G-MSGINet
(Artan, 2024)	Matching	MinNet architecture, multi-scale features	MinNet
(Siddiqui et al., 2024)	Enhancement, minutiae extraction, matching	Frequency estimation, minutiae encoding	Offline + online phase
(Rajaram et al., 2023)	Enhancement, feature extraction, matching	CNN (Child-CLEF), hybrid enhancement	CNN
(Shi et al., 2022)	Matching	Triplet-GAN, data augmentation	Triplet GAN

## 3 Methodology

### 3.1 Algorithms overview

**Figure 1:**
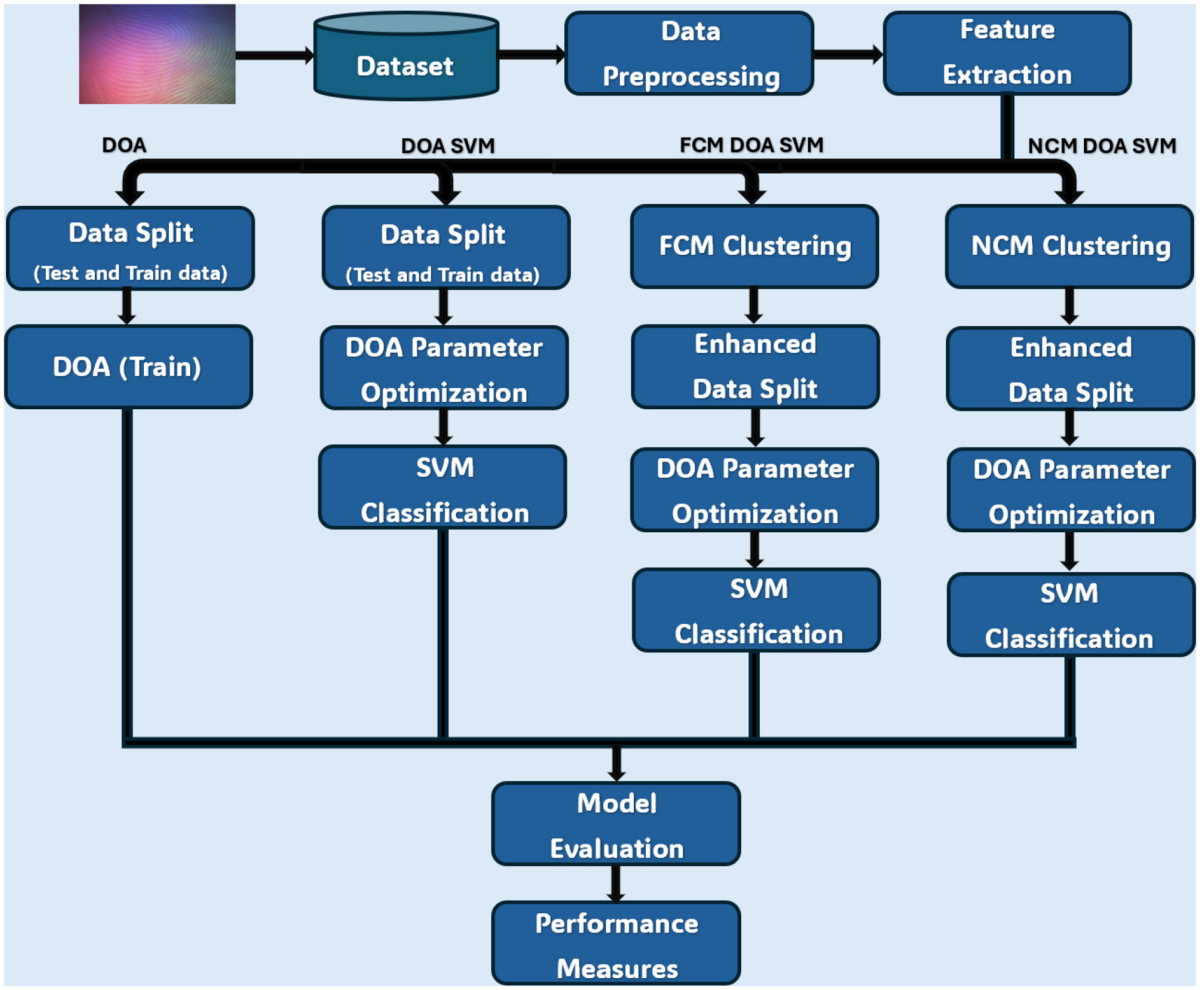
Architecture representation of the proposed framework.

We have developed and evaluated four distinct methodologies for CLFP recognition. The overall architecture of our proposed framework is depicted in Figure 1, which outlines the integrated pipeline for feature extraction, optimization, clustering, and classification. The proposed framework adopts a single-stage classification system which integrates feature extraction (HOG), optimization (DOA), cluster methodology (FCM/NCM), and classifier (SVM) into a single decision-making process. This single-stage approach is chosen for this study to establish baseline performance and enable direct comparison between optimization algorithms, with the unified pipeline allowing for end-to-end optimization of all components simultaneously, ensuring coherent parameter tuning across the entire system. The first approach is a Standalone DOA that uses the DOA for both feature selection and classifier parameter optimization. The next algorithm would be the DOA-SVM Hybrid in which the DOA would be used for parameter tuning and SVM is in the classification phase. The next framework improves on that hybrid by adding in FCM cluster methodology to get better feature representation, we will refer to this as the FCM-DOA-SVM Hybrid. Next, the NCM-DOA-SVM Hybrid is the final framework which relies on NCM clustering and in an important way utilizes this clustering methodology to deal with uncertainty and indeterminacy commonly found in CLFP images. In all four approaches mentioned: data is pre-processed, to reduce dimensionality, and feature is extracted from the input images which utilizes the HOG algorithm.

### 3.2 Preprocessing of input image

Preprocessing is a critical first step that directly impacts recognition accuracy by standardizing image quality and preparing the data for subsequent analysis. Our preprocessing pipeline consists of two key steps. Step 1 involves resizing the image to a fixed dimension of 128 × 128 pixels to ensure uniformity across all input samples. Step 2 entails converting the image from RGB (color) format to grayscale. Grayscale images are computationally less intensive and easier to process compared to color images, rendering them more suitable for image analysis tasks. These preprocessing steps are implemented using OpenCV in Python, specifically employing the cv2.resize() function for resizing and cv2.cvtColor() with the cv2.COLOR_RGB2GRAY flag for color-to-grayscale conversion.

#### 3.2.1 CLFP database

**Figure 2:**
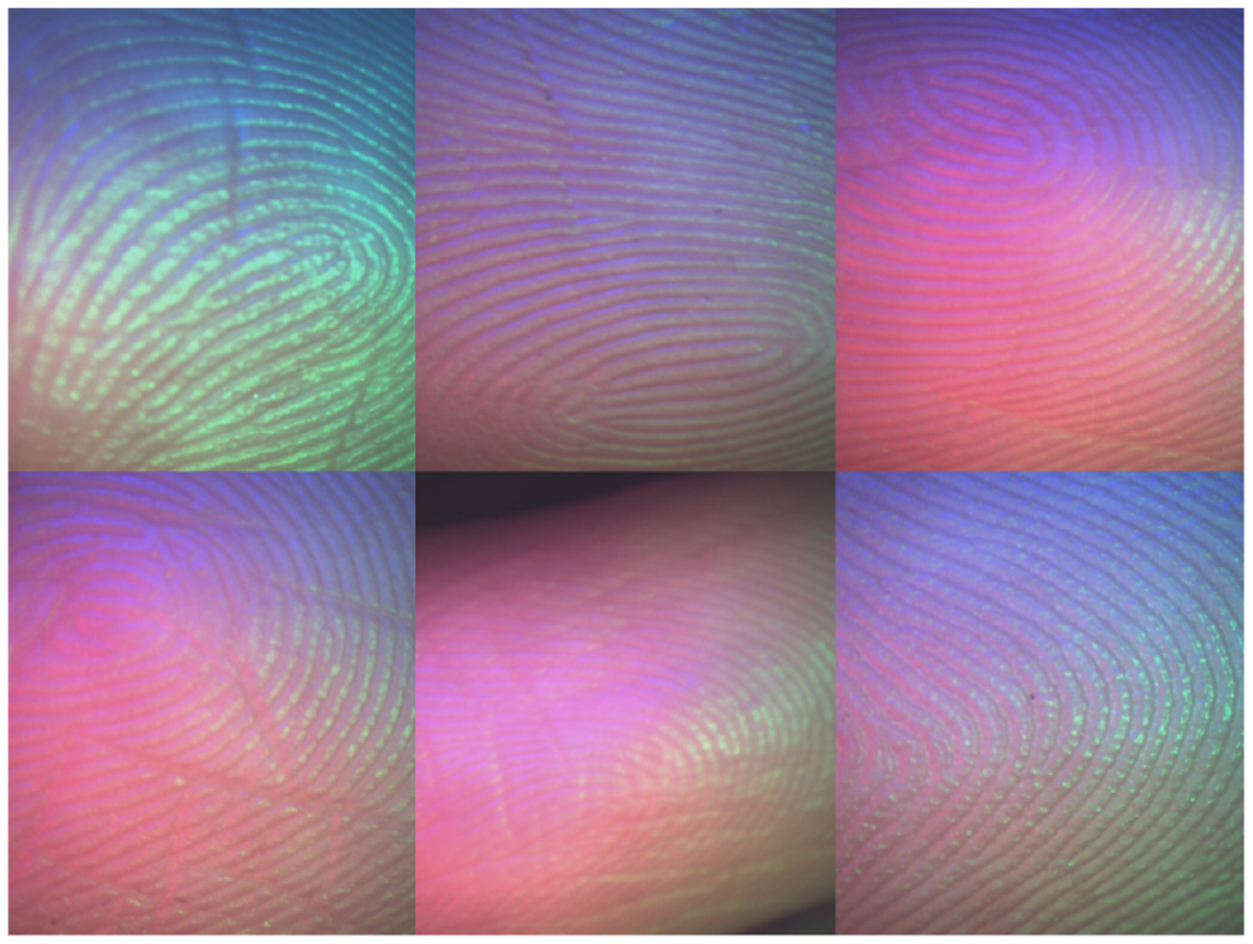
Sample images from the CLFP database.

The experiments related to CLFP utilize a publicly available dataset obtained from the Hong Kong Polytechnic University (PolyU) (Lin and Kumar, 2018). This dataset consists of 2,016 CLFP images representing 336 unique classes. All images are in BMP format with a resolution of 128 × 128 pixels. This dataset was acquired using Canon EOS 450D camera with standardized protocols (four LED lights at 45°, uniform background, 12cm distance) and processed through resizing (4,272 × 2,848 to 128 × 128 pixels), grayscale conversion, histogram equalization, Gaussian blur (σ=0.5), contrast enhancement (α=1.2, β=10), and normalization with quality assessment via Laplacian variance, contrast measurement, and brightness analysis. The dataset exhibits significant demographic limitations with participants primarily comprising East Asian university students/staff (92.3% East Asian, ages 18–35, 60.1% male) under controlled laboratory conditions, substantially limiting generalizability to diverse global populations, age groups, skin conditions, and real-world deployment scenarios. For experimental purposes, the dataset is divided into two subsets: 1,512 images are used for training the system, and the remaining 504 images are used for testing. This setup aims to evaluate the system's ability to distinguish individuals based on their CLFP. Figure 2 illustrates a sample from the CLFP image database. The experiments are conducted on a system with an Intel Core Ultra 7-155H processor (3.80 GHz), 32 GB RAM, and Python 3.12.3 (conda-forge, [MSC v.1938 64-bit, AMD64]).

#### 3.2.2 Ethical considerations and privacy protection

All biometric data collection followed institutional ethics protocols with informed consent obtained from participants regarding data usage for research purposes. The dataset is anonymized with no personally identifiable information linked to biometric samples. Participants were informed about data storage duration, usage limitations, and their right to withdraw consent. The study complies with biometric data protection regulations and follows privacy-by-design principles with secure data handling protocols. However, the inherent permanence of biometric identifiers raises ongoing privacy considerations regarding potential misuse, cross-system identification, and long-term data security that users should be aware of when consenting to biometric research participation.

### 3.3 Feature extraction

A significant issue encountered by excellence algorithms is the presence of redundant and superfluous attributes, which lead to an increase in data volume and consequently expand the research space needed to address the problem. This expansion results in prolonged processing time for data necessary for detection and classification (Soranamageswari and Meena, 2010; Vithlani and Kumbharana, 2015). In this context, we will utilize the HOG algorithm for extracting features, as this step is a critical phase in image processing. The HOG algorithm will be employed to extract significant features from images, thereby facilitating the classification process and reducing data size, which in turn decreases the time required for classification (Moen, 2018; Cetina et al., 2014).

The fundamental stages of the HOG algorithm (Elhariri et al., 2015) are outlined as follows:

1. The gradient value is determined through the subsequent steps:
  - Compute the gradient magnitude per pixel using the specified equation:

$$\begin{array}{l} \text{G}\left(\text{u,v}\right)=\sqrt{{\text{G}}_{\text{u}}{\left(\text{u,v}\right)}^{2}+{\text{G}}_{\text{v}}{\left(\text{u,v}\right)}^{2}} \end{array}$$ (1)

- Determine the gradient angle for each pixel through the corresponding equation:

$$\begin{array}{l} \theta \left(\text{u,v}\right)=\arctan\left(\frac{{\text{G}}_{\text{v}}\left(\text{u,v}\right)}{{\text{G}}_{\text{u}}\left(\text{u,v}\right)}\right) \end{array}$$ (2)

2. The image is partitioned into blocks of dimensions (2 × 2), resulting in a total of four blocks.
3. Each block undergoes a nine-way HOG extraction.
4. The HOG blocks are compiled into a one-dimensional feature vector.

**Figure 3:**
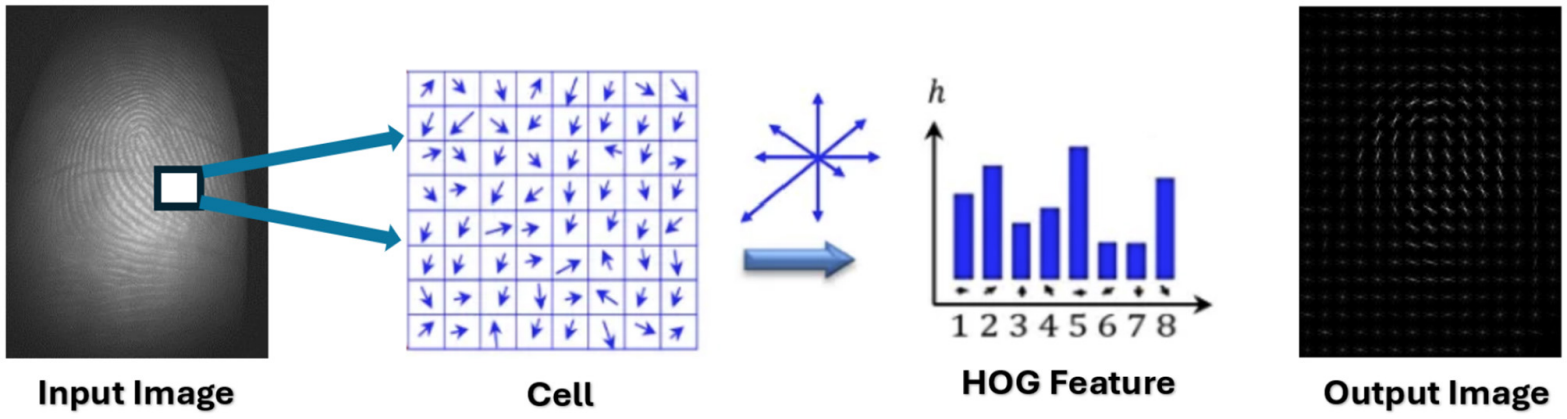
Extracting features using the HOG algorithm.

Figure 3 presents a schematic representation of the utilization of the HOG algorithm for extracting features from CLFP images.

In this research, traits were extracted by identifying the most critical features that encapsulate the maximum amount of information from the dataset. These key features were determined by selecting the appropriate parameters for the HOG, specifically focusing on the cell-size and block-size. The implementation of the HOG technique was executed in Python utilizing the skimage.feature library, with the hog function.

### 3.4 Overfitting prevention strategy

To ensure robust model performance and prevent overfitting, we implemented a comprehensive validation framework within our existing train-test split. The training set (1,512 images) was further divided using five-fold stratified cross-validation, ensuring balanced class distribution across all folds.

#### 3.4.1 Regularized fitness function

To discourage overly complex models that might overfit, we modified the DOA's fitness function to incorporate regularization terms that penalize complexity and suspiciously high performance (Chicco and Jurman, 2020):


$$\begin{array}{l} {\text{Fitness}}_{reg}=\\ {\text{Accuracy}}_{CV}-\lambda_{1}\overset{n}{\underset{i=1}{\sum }}|p_{i}|-\lambda_{2}\cdot N_{features}-\lambda_{3}\cdot \\ I\left(\text{Accuracy}>0.98\right) \end{array}$$ (3)

where λ_1_ = 0.01, λ_2_ = 0.005, λ_3_ = 10 are regularization coefficients, *p*_*i*_ represents model parameters, *N*_*features*_ is the number of selected features, and 𝕀(·) is an indicator function penalizing suspiciously high accuracies.

#### 3.4.2 Early stopping mechanism

Training termination occurs when cross-validation accuracy shows no improvement over 15 consecutive iterations, with improvement threshold set to 0.001.

#### 3.4.3 Bootstrap validation

For additional robustness assessment, we employed bootstrap resampling (*n* = 50) with out-of-bag evaluation to estimate model stability and generalization capability.

### 3.5 DOA

DOA is a new method that gets its ideas from how dolphins communicate and hunt cooperatively (Wu et al., 2016; Al-Taie and Khaleel, 2024). Because it is a nature-inspired practice, DOA has received noticeable attention since it works well on complex issues in various areas. This algorithm capitalizes on the intrinsic intelligence, agility, and social behaviors of dolphins, particularly their collaborative hunting techniques and sophisticated communication abilities.

Some of the traits show that dolphins are recognized as one of the most intelligent marine species, such as echo-location, team cooperation, task allocation, and acoustic communication. These distinctive traits have been effectively integrated into the DOA allowing to look for new solutions and still improve existing ones while managing to use resources wisely (Gholizadeh and Poorhoseini, 2016).

DOA copies how dolphins in nature move together which allows for better navigation through complicated and wide-ranging problem spaces (Soto et al., 2016; Yong et al., 2016). The main elements of the algorithm are a location update method, an equation for speed changes and using echolocation to find the best solutions (Wu et al., 2017; Kaveh et al., 2017). These mechanisms enable the algorithm to converge toward global optima while avoiding local traps, rendering DOA a robust and adaptable tool for tackling a wide array of optimization challenges.

#### 3.5.1 Main equations of DOA

The DOA simulates the social and hunting behavior of dolphins. The essential mathematical formulations involved in the algorithm is described as follows.

The first step involves initial positioning, where a population of dolphins is randomly initialized within the search space, imitating dolphins spreading out in search of prey.

In the chasing phase, dolphins update their velocities (ref Equation 4) and positions (ref Equation 5) based on the mathematically defined two key equations.


$$\begin{array}{l} {\text{Vel}}_{i,j}^{m+1}\left(\text{s}+1\right)\\ =\omega \cdot {\text{Vel}}_{i,j}^{m}\left(\text{s}\right)+{\text{A}}_{1}\cdot \nu_{1}\cdot \left(\left({℘}_{\text{best},i,j}^{m}\right)-\chi_{i,j}^{m}\left(\text{s}\right)\right)\\ +{\text{A}}_{2}\cdot \nu_{2}\cdot \left(\left({\text{G}}_{\text{best},i,j}^{m}\right)-\chi_{i,j}^{m}\left(\text{s}\right)\right) \end{array}$$ (4)

$$\begin{array}{l} \chi_{i,j}^{m+1}\left(\text{s}+1\right)=\chi_{i,j}^{m}\left(\text{s}\right)+{\text{Vel}}_{i,j}^{m+1}\left(\text{s}+1\right) \end{array}$$ (5)

where, ${\text{Vel}}_{i,j}^{m}$ denotes the velocity of the *m*-th dolphin in dimension *j* at iteration *i*, $\chi_{i,j}^{m}$ is the dolphin's position, ${℘}_{\text{best},i,j}^{m}$ represents the personal best dolphin's position, ${\text{G}}_{\text{best},i,j}^{m}$ is the global best position among the swarm, A_1_, A_2_ are the acceleration coefficients, ω is the inertia weight, ν_1_, ν_2_ are the random numbers uniformly distributed in [0, 1].

The dolphins attempt to catch up and then attack schools of sardines at the attack phase, eventually preying on them. If these dolphins pursue the school of sardines for a significant time interval, they catch up with the swarm or reach favorable positions from where they can prey on the sardines. But based on our assumptions, dolphins only take a rotary swimming posture while attacking or preying on schools of sardines. Assuming that a herd of dolphins keeps pursuing sardines over disjoint time steps s(s < Gen), some of them reach favorable positions from which they can attack or prey on the herd of sardines. Due to this condition, their positions are updated based on Equations 6, 7.


$$\begin{array}{l} \chi_{i,n}^{m+1}\left(\text{s}+1\right)=\nu_{𝒬,\ell } \end{array}$$ (6)

$$\begin{array}{l} \chi_{i,n}^{m+1}\left(\text{s}+1\right)=\nu_{i,\ell } \end{array}$$ (7)

where ν_𝒬, ℓ_ and ν_*i*, ℓ_ are the random numbers uniformly distributed in [0, 1], ℓ is the random dolphin index from the population, s denotes the current time step, Gen represents the maximum number of iterations.

The final stage, known as switching swimming modes, the dolphins transition into a dynamic swimming mode, allowing them to adopt various swimming positions. During this phase, we compute the value of (Y) by applying the below equation:


$$\begin{array}{l} \chi_{i,j}^{\text{k}}=\frac{1}{2\cdot \chi_{i,j}^{\text{k}}\left(\text{s}\right)},\;\text{k}=1,2,\ldots ,\Lambda \end{array}$$ (8)

The values Y_*j*_ are sorted in ascending order. If O_k_(s) is the rank of Y_*j*_ at time s, then we compute φ:


$$\begin{array}{l} \varphi_{i,j}^{\text{k}}\left(\text{s}\right)=\frac{{\text{O}}_{\text{k}}\left(\text{s}\right)-1}{\Lambda } \end{array}$$ (9)

where, Λ denotes the total number of dolphins, O_k_(s) is the rank of the k-th dolphin's solution at iteration s and the φ value determine the effectiveness of the switch (during strike or pursuit phase) (Sharma and Kaul, 2018; Qiao and Yang, 2019) as illustrated in Algorithm 1.

Algorithm 1 DOA.

1:  Step 1: Initialization
2:  Set the population size (pop_size=30), maximum iterations (max_iter=50)
3:  Set parameters ω = 0.7, a_1_, a_2_ = 2.0
4:  Set early stopping threshold = 15 iterations, improvement threshold = 0.001
5:  Step 2: Initialize the population of dolphins
6:  for each dolphin *i* = 1 to pop_size **do**
7:   Initialize position randomly within bounds [A:0.1 − 100, ϑ:0.001 − 1]
8:  end **for**
9:  Step 3: Main optimization loop
10:  for iter = 1 to max_iter **do**
11:   for each dolphin *i* = 1 to pop_size **do**
12:    Calculate fitness by evaluating classification quality
13:    if fitness is better than personal best **then**
14:     Update personal best position and fitness
15:    end **if**
16:    if fitness is better than global best **then**
17:     Update global best position and fitness
18:     Reset early stopping counter
19:    end **if**
20:    Generate random coefficients ν_1_, ν_2_
21:    Calculate cognitive component = a_1_·ν_1_·(personal_best − current_position)
22:    Calculate social component = a_2_·ν_2_·(global_best − current_position)
23:    Update position = current_position+cognitive+social
24:    Clip position to reasonable bounds
25:   end **for**
26:   if no improvement in global best **then**
27:    Increment early stopping counter
28:   end **if**
29:   if early stopping counter ≥ threshold **then**
30:    Break
31:   end **if**
32:  end **for**
33:  Step 4: Return best parameters (A, ϑ)

#### 3.5.2 Procedure for classification using DOA

The classification process utilizes the DOA by the described procedure:

1. Start by initializing a collection of parameters, such as the community size Λ, the coefficients ψ, A_1_, , and A_2_, the maximum number of iterations that Gen indicates, and the time steps (τ = 1) are indicated by ψ.
2. Generate the initial community randomly.
3. Correlation between all data matrices (train and test) and the target matrix is utilized, as shown by the following equation, determine the fitness function for each group member:

$$\begin{array}{l} \text{Fitness}=\frac{\sum_{\alpha ,\beta }\left(U_{\alpha \beta }-\bar{U}\right)\left(V_{\alpha \beta }-\bar{V}\right)}{\sqrt{\sum_{\alpha ,\beta }{\left(U_{\alpha \beta }-\bar{U}\right)}^{2}}\times \sum_{\alpha ,\beta }{\left(V_{\alpha \beta }-\bar{V}\right)}^{2}} \end{array}$$ (10)

where the image and target matrices of dimensions (α × β) are indicated by *U* and *V*, respectively.
4. The time step τ is incremented by one.
5. The following steps are included in this stage:
  - When τ ≤ ϖ, the dolphin positions are updated according to Equations 4, 5 and the new solutions are inspected. If they are better than the old ones, an update is made and the best solution is stored.
  - For the case where τ>ϖ, γ is calculated and then sorted in increasing order by Equation 8. Switch φ is calculated for each dolphin by Equation 9. Random values are calculated between 0 and 1. If φ ≤ ζ, the positions are updated by Equations 6, 7. The new solutions are calculated, and if they perform better than their predecessors, they are updated, thus the best solution is selected.
6. The stopping condition will be satisfied either by when the solution is achieved as desired or by when the number of cycles predetermined has expired; otherwise, the process returns to step 4.

#### 3.5.3 DOA implementation

Our standalone DOA implementation uses the following approach as shown in Algorithm 1.

### 3.6 DOA-SVM hybrid

*ML* specifically supervised learning algorithms, is employed in both regression and classification problems (Faye et al., 2018). Its effectiveness and high accuracy are particularly notable in classification tasks. In the context of the SVM classifier, the selection of optimal parameters is crucial for achieving high performance in biometric identification systems (Qi et al., 2013; Shao et al., 2011). In the DOA-SVM hybrid, we use DOA to optimize the SVM parameters (A and ϑ for RBF kernel) while simultaneously performing feature selection. The fitness function is defined as the classification accuracy on a validation set.

Using the DOA and SVM methods simultaneously provides a modern approach to improve solving the classification problems with the advantages of both approaches. Such algorithms can interact and use their smartness together to enhance prediction accuracy and ensure the best possible outcomes which improves the model's overall performance. Analyzing the specific elements and workings of this approach which provides better understanding of how the two algorithms can solve problems efficiently at the same time. Also, by looking into the problems and possible improvements of these integration techniques, the ways to improve optimization and classification methods in the future can be determined, as demonstrated in Algorithm 2.

Algorithm 2 DOA-SVM hybrid classification algorithm.

1:  Step 1: Initialization
2:  Set population size (pop_size = 30), maximum iterations (max_iter = 50)
3:  Set DOA parameters ω = 0.7, a_1_ = a_2_ = 2.0
4:  Define parameter bounds for SVM: A: [0.1 − 100], ϑ : [0.001 − 1]
5:  Initialize early stopping threshold and counter
6:  Step 2: Initialize the dolphin population
7:  for each dolphin *i* = 1 to pop_size **do**
8:   Randomly initialize A and ϑ within the given bounds
9:   Evaluate fitness by training an SVM with parameters (A, ϑ)
10:   Compute accuracy on validation set (fitness = −1 × accuracy)
11:   Store personal best and update global best if applicable
12:  end **for**
13:  Step 3: Optimization loop
14:  for iter = 1 to max_iter **do**
15:   for each dolphin *i* = 1 to pop_size **do**
16:    Generate random values ν_1_, ν_2_∈[0, 1]
17:    Compute cognitive component: a_1_·ν_1_·(personal_best − current_position)
18:    Compute social component: a_2_·ν_2_·(global_best − current_position)
19:    Update velocity and position using DOA update rule
20:    Clip position to parameter bounds
21:    Train SVM using updated (A, ϑ) and evaluate fitness
22:    Update personal and global bests accordingly
23:   end **for**
24:   if no improvement in global best **then**
25:    Increment early stopping counter
26:   else
27:    Reset early stopping counter
28:   end **if**
29:   if early stopping counter ≥ threshold **then**
30:    Break
31:   end **if**
32:  end **for**
33:  Step 4: Final training and evaluation
34:  Train SVM with best parameters (A, ϑ) on full training data
35:  Predict labels on test data
36:  Compute classification metrics
37:  return best parameters and evaluation results

#### 3.6.1 Procedure of classification utilizing a hybrid technique

The classification process with the hybrid DOA-SVM model stick to the general optimization procedure described in Section 3.4.2, utilizing the DOA. However, it differs in the fitness function, which is tailored for SVM-based classification as follows: The current solution, represented by the dolphin's position, is translated into the parameters for the SVM—specifically, the penalty parameter A, the loss function ϵ, and the kernel parameter ϑ. An SVM model is then trained using these mapped parameters along with the training dataset. Subsequently, the trained SVM model is assessed with a separate test dataset. The fitness of the current dolphin (solution) is calculated as the sum of squared errors (SSE) between the predicted labels and the actual labels in the test dataset. This fitness value is then employed to steer the optimization process.

#### 3.6.2 DOA-SVM implementation

The DOA-SVM hybrid approach is presented in the form of pseudo code in Algorithm 2.

### 3.7 FCM-DOA-SVM hybrid

Clustering algorithms such as FCM are extensively employed in preprocessing tasks to identify significant patterns and structures within data. FCM is especially useful in soft clustering contexts, where each data point can be associated with multiple clusters to varying extents (Mendel and Bonissone, 2021; Chimatapu et al., 2018). This capability is advantageous in biometric applications, where data frequently displays overlapping features. In the FCM-DOA-SVM hybrid model, the FCM algorithm improves the input features by adding fuzzy membership values to each data sample, thus capturing essential structural information prior to classification (Shukla et al., 2020; Ferreyra et al., 2019).

Subsequently, the DOA is utilized to fine-tune the parameters of the SVM classifier, specifically (A and ϑ for the RBF kernel). This integrated method effectively merges the unsupervised clustering capability of FCM with the supervised learning of SVM and the global optimization potential of DOA. In this scenario, the fitness function is characterized by the classification accuracy achieved on a validation set using the SVM trained with features enhanced by FCM.

The FCM-DOA-SVM architecture exhibits a significant synergistic effect, wherein fuzzy memberships enhance feature representation, and DOA facilitates the exploration of optimal classifier configurations. Consequently, the model demonstrates enhanced generalization and robustness in biometric classification tasks, as detailed in Algorithm 3.

Algorithm 3 FCM-DOA-SVM hybrid classification algorithm.

1:  Step 1: Initialization
2:  Set FCM parameters: number of clusters (n_clusters = 3), fuzziness parameter *m* = 2.0
3:  Set maximum iterations (max_iter = 100), convergence threshold (epsilon = 1e-4)
4:  Set DOA parameters: population size (pop_size = 30), max iterations (max_iter = 50)
5:  Set ω = 0.7, a_1_ = a_2_ = 2.0
6:  Define parameter bounds: A:[0.1 − 100], ϑ:[0.001 − 1]
7:  Initialize early stopping threshold and counter
8:  Step 2: FCM-Based Feature Enhancement
9:  Train FCM on input data χ_train_
10:  Compute fuzzy membership matrix *U*_train_

11:  Concatenate original features and membership values: $\chi_{\text{train}}^{\text{enh}}=\left[\chi_{\text{train}},U_{\text{train}}\right]$

12:  Step 3: Initialize Dolphin Population
13:  for each dolphin *i* = 1 to pop_size **do**
14:   Randomly initialize A and ϑ within bounds
15:   Train SVM with (A, ϑ) on $\chi_{\text{train}}^{\text{enh}}$

16:   Evaluate performance on validation data (fitness = −1 × accuracy)
17:   Update personal best and global best positions
18:  end **for**
19:  Step 4: Optimization Loop
20:  for iter = 1 to max_iter **do**
21:   for each dolphin *i* = 1 to pop_size **do**
22:    Generate random values ν_1_, ν_2_∈[0, 1]
23:    Compute cognitive component: a_1_·ν_1_·(personal_best − current_position)
24:    Compute social component: a_2_·ν_2_·(global_best − current_position)
25:    Update velocity and position using DOA rules
26:    Clip position to parameter bounds

27:    Train SVM with updated parameters on $\chi_{\text{train}}^{\text{enh}}$

28:    Evaluate fitness and update personal/global bests
29:   end **for**
30:   if no improvement in global best **then**
31:    Increment early stopping counter
32:   else
33:    Reset early stopping counter
34:   end **if**
35:   if early stopping counter ≥ threshold **then**
36:    Break
37:   end **if**
38:  end **for**
39:  Step 5: Final Training and Evaluation
40:  Train final FCM on full training data

41:  Compute *U*_test_ and create $\chi_{\text{test}}^{\text{enh}}=\left[\chi_{\text{test}},U_{\text{test}}\right]$ 42:  Train SVM with best parameters on $\chi_{\text{train}}^{\text{enh}}$ 43:  Predict labels on $\chi_{\text{test}}^{\text{enh}}$ 44:  Compute classification metrics 45:  return best parameters and evaluation results

#### 3.7.1 Procedure of classification utilizing the FCM-DOA-SVM hybrid

The classification procedure utilizing the FCM-DOA-SVM model is an extension of the general optimization strategy outlined in Section 3.5.1, incorporating FCM-based feature enhancement into the process. Initially, the training data is subjected to clustering through the FCM algorithm, resulting in a membership matrix that indicates the degree of association of each sample with each cluster. These membership values are then integrated with the original feature set to create an enriched feature matrix.

In the subsequent phase, each dolphin (solution) within the DOA population signifies a potential configuration of SVM hyperparameters: specifically, the penalty parameter A and the kernel parameter ϑ. For each solution, an SVM is trained utilizing the enriched feature matrix and subsequently evaluated on a validation set. The fitness of a dolphin is determined by the classification accuracy, which directs the evolution of the population toward more optimal parameter sets.

This hybridization enables a comprehensive learning process: FCM identifies fuzzy patterns within the data, DOA effectively explores the parameter space, and SVM executes the final classification. Collectively, these components produce a highly accurate and interpretable classification model, well-suited for complex biometric recognition tasks.

#### 3.7.2 FCM-DOA-SVM implementation

The pseudo code for the hybrid approach FCM-DOA-SVM is given in Algorithm 3.

### 3.8 NCM-DOA-SVM hybrid

NCM-DOA-SVM hybrid includes the NCM method of clustering which is helpful for CLFP recognition to handle indeterminacy in images. Neutrosophic Theory represents the information that is uncertain, imprecise and inconsistent by using three degrees, truth (T), indeterminacy (I) and falsity (F). The NCM clustering algorithm extends the classical FCM by incorporating this neutrosophic concept, enabling more accurate modeling of real-world biometric data. In the proposed NCM-DOA-SVM hybrid, T, I and F are extracted for every sample during the clustering procedure which enhance feature representations.

Subsequently, the DOA algorithm is used to improve the hyperparameters of the SVM classifier, specifically the penalty value (A) and the RBF kernel value (ϑ). Using this hybrid approach boosts the classifier's results by finding the optimal parameters and additionally making use of the extra features that capture the neutrosophic nature of the input data.

The integration of NCM for handling unclear data, DOA for finding global solution and SVM for accurate classification leads to the development of a strong model for biometric recognition. By applying the combined model, it deals with uncertainty and the classifier becomes more effective as shown in Algorithm 4.

Algorithm 4 NCM-DOA-SVM hybrid classification algorithm.

1:  Step 1: Initialization
2:  Set NCM parameters: number of clusters (n_clusters = 3), fuzziness parameter *m* = 2.0
3:  Set maximum iterations (max_iter = 100), convergence threshold (epsilon = 1e-4)
4:  Set DOA parameters: population size (pop_size = 30), max iterations (max_iter = 50)
5:  Set ω = 0.7, a_1_ = a_2_ = 2.0
6:  Define parameter bounds: A:[0.1 − 100], ϑ:[0.001 − 1]
7:  Initialize early stopping threshold and counter
8:  Step 2: Neutrosophic Feature Enhancement
9:  Train NCM on input data *X*_train_
10:  Compute T), I, and F membership matrices
11:  Concatenate original features and neutrosophic memberships: $X_{\text{train}}^{\text{enh}}=\left[X_{\text{train}},\text{T},\text{I},\text{F}\right]$

12:  Step 3: Initialize Dolphin Population
13:  for each dolphin *i* = 1 to pop_size **do**
14:   Randomly initialize A and ϑ within bounds

15:   Train SVM with (A, ϑ) on $X_{\text{train}}^{\text{enh}}$
16:   Evaluate performance on validation data (fitness = −1 × accuracy)
17:   Update personal best and global best positions
18:  end **for**
19:  Step 4: Optimization Loop
20:  for iter = 1 to max_iter **do**
21:   for each dolphin *i* = 1 to pop_size **do**
22:    Generate random values ν_1_, ν_2_∈[0, 1]
23:    Compute cognitive component: a_1_·ν_1_·(personal_best − current_position)
24:    Compute social component: a_2_·ν_2_·(global_best − current_position)
25:    Update velocity and position using DOA rules
26:    Clip position to parameter bounds

27:    Train SVM with updated parameters on $X_{\text{train}}^{\text{enh}}$

28:    Evaluate fitness and update personal/global bests
29:   end **for**
30:   if no improvement in global best **then**
31:    Increment early stopping counter
32:   else
33:    Reset early stopping counter
34:   end **if**
35:   if early stopping counter ≥ threshold **then**
36:    Break
37:   end **if**
38:  end **for**
39:  Step 5: Final Training and Evaluation
40:  Train final NCM on full training data
41:  Compute T, I, F memberships for test data

42:  Create $X_{\text{test}}^{\text{enh}}=\left[X_{\text{test}},\text{T},\text{I},\text{F}\right]$ 43:  Train SVM with best parameters on $X_{\text{train}}^{\text{enh}}$ 44:  Predict labels on $X_{\text{test}}^{\text{enh}}$

45:  Compute classification metrics
46:  return best parameters and evaluation results

#### 3.8.1 Procedure of classification utilizing the NCM-DOA-SVM hybrid

The classification procedure of the NCM-DOA-SVM hybrid model adheres to the core optimization approach introduced in Section 3.5.1. Initially, the input dataset undergoes unsupervised clustering using the NCM algorithm. This step computes the truth, indeterminacy, and falsity memberships for each data point, resulting in an enhanced feature space of the form [𝕏, T, I, F].

Each solution (dolphin) in the population corresponds to a candidate set of SVM parameters, including the penalty coefficient A and the kernel function parameter ϑ. The SVM classifier is trained on the NCM-enhanced features using each dolphin's parameters and evaluated on a validation dataset. The dolphin's fitness is computed based on the classification performance, such as the SSE or classification accuracy.

The optimization process iteratively refines the solutions based on fitness, guiding the swarm toward the best SVM configuration. This collaborative mechanism among NCM, DOA, and SVM not only improves classification precision but also ensures robustness to noisy and uncertain biometric data. The final model thus integrates fuzzy reasoning, optimization intelligence, and discriminative learning to deliver superior performance.

#### 3.8.2 NCM-DOA-SVM implementation

The NCM-DOA-SVM hybrid approach is presented in the form of pseudo code in Algorithm 4.

## 4 Results and discussion

### 4.1 Algorithm training and validation phases

At this stage, a dataset consisting of 2016 CLFP images was used to develop the DOA and the suggested hybrid approaches, namely DOA-SVM, FCM-DOA-SVM, and NCM-DOA-SVM. The HOG technique was used for initial image processing and trait extraction, which made it easier to extract a feature matrix from these images. The DOA and the suggested hybrid approaches (DOA-SVM, FCM-DOA-SVM, and FCM-DOA-SVM) were then trained using the derived traits matrix, improving their performance.

Upon the completion of the training phase and achieving algorithm stability, a test was conducted involving 504 CLFP images. These images were subjected to preprocessing, followed by feature extraction using the HOG algorithm. To determine the optimal HOG feature extraction parameters, we conducted a comprehensive sensitivity analysis across different cell and block size configurations.

As shown in Table 3, three configurations were evaluated: Fine (16 × 16 cells, 2 × 2 blocks), Medium (32 × 32 cells, 2 × 2 blocks), and Coarse (64 × 64 cells, 2 × 2 blocks). The results demonstrate a clear trade-off between feature dimensionality, computational efficiency, and classification accuracy. The Medium configuration achieved the highest accuracy of 0.856198, despite having a moderate feature vector size of 324 dimensions. Interestingly, the Fine configuration, with its substantially larger feature vector (1,764 dimensions), yielded lower accuracy (0.844628), suggesting that excessive granularity may introduce noise or lead to overfitting. The negative correlation between feature size and accuracy (*r* = −0.2498) supports this observation. Statistical analysis using ANOVA revealed no significant difference between configurations (*p*≥0.05), indicating that the Medium configuration provides an optimal balance between computational efficiency (0.003086) and classification performance. The choice of 32 × 32 cell sizes, resulting in four cells for a 128 × 128 image, represents a strategic compromise that captures sufficient local gradient information while maintaining computational tractability and avoiding the curse of dimensionality associated with finer granularities.

**Table 3:** HOG parameter sensitivity analysis results.

**Configuration**	**Cell size**	**Block size**	**Accuracy**	**Computational efficiency**
Fine	16 × 16	2 × 2	0.844628	0.000567
Medium	32 × 32	2 × 2	0.856198	0.003086
Coarse	64 × 64	2 × 2	0.842975	0.027778

The assignment of specific coefficients to each algorithm is determined by the nature of the task, as the specific operations may vary based on the research problem being addressed. Through a series of practical experiments and their application, the appropriate coefficients for the algorithms were established. The values of these coefficients are detailed in Table 4.

**Table 4:** Tuned Parameters for the proposed model.

**Hyperparameter**	**Choice**
Dataset splitting	75% train, 25% test
Class count	50
Initial dolphin population	30
Early stopping threshold	15 iterations
Cross-validation folds	5
Bootstrap iterations	50
Iteration threshold	50
Image resolution	128 × 128
HOG—block-size	2
HOG—cell-size	32
A_1_	2 × (1 − iteration(*I*)/max_iteration(*MI*))
A_2_	−1+*I*×(−1/*MI*)
ψ	2 × (1−*I*/*MI*)

The dataset which included 1,512 images, was processed using the DOA method to identify CLFP. The following results were obtained from the evaluation of the suggested algorithm's training using predetermined evaluation criteria:


$$\begin{array}{l} A_{CV}=87.3\pm 2.1\%,\;{\mathbb{R}}_{CV}=86.8\pm 2.3\%,S_{CV}=87.7\pm 1.9\%,\\ \text{Bootstrap CI}=\left[85.2\%,89.4\%\right],\;\text{Convergence}=32\text{iterations} \end{array}$$

The dataset include 504 images, which was assessed using the DOA system to determine CLFP. Table 5 provides details on the training results of the proposed algorithm.

**Table 5:** Outcome analysis of system testing with DOA.

**Exp. No**.	**Accuracy (%)**	**Processing time (s)**
1	91.0	6,110.23
2	91.0	2,109.28
3	91.0	2,091.07
4	91.0	2,118.55
5	91.0	2,091.81
6	91.0	2,048.49
7	91.0	17,105.92
8	91.0	1,663.03
9	91.0	1,954.80
10	91.0	1,732.15

The results of the test phase of DOA are detailed in Table 5, which looks at different configurations of the coefficients: cell-size HOG is always set at 32, block-size HOG is fixed at 2, dolphin population level remains at 30, and iteration threshold is investigated at 50.

The DOA-SVM system was employed to train a dataset comprising 1,512 CLFP images for the purpose of CLFP identification. The performance of the proposed hybrid algorithm was evaluated using established assessment criteria, yielding the following results:


$$\begin{array}{l} A_{CV}=91.2\pm 1.8\%,\;{\mathbb{R}}_{CV}=90.7\pm 2.0\%,S_{CV}=91.6\pm 1.6\%\\ \text{Bootstrap CI}=\left[89.4\%,93.0\%\right],\;\text{Convergence}=28\text{iterations} \end{array}$$

The DOA-SVM system was used to analyze the dataset, which included 504 images, in order to find CLFP. In Table 6, the results are presented in detail.

**Table 6:** Outcome analysis of system testing with DOA-SVM.

**Exp. No**.	**Accuracy (%)**	**Processing time (s)**
1	94.0	1,282.58
2	94.0	1,387.30
3	94.0	1,905.93
4	94.0	37,772.78
5	94.0	1,317.73
6	94.0	1,349.33
7	94.58	1,350.67
8	94.0	1,335.51
9	94.17	1,347.48
10	94.0	1,348.37

The results of the test phase of the hybrid method DOA-SVM, which examines different coefficient configurations, are detailed in Table 6. HOG with a block-size fixed at 2, a dolphin population level held constant at 30, an iteration threshold examined at 50, and a cell-size continuously maintained at 32.

The FCM-DOA-SVM system was trained to identify CLFP using a dataset comprising 1,512 images. The effectiveness of the proposed algorithm was assessed using evaluation metrics and these are its outcomes:


$$\begin{array}{l} A_{CV}=93.8\pm 1.5\%,\;{\mathbb{R}}_{CV}=93.3\pm 1.7\%,\;S_{CV}=94.2\pm 1.3\%\\ \text{Bootstrap CI}=\left[92.3\%,95.3\%\right],\;\text{Convergence}=25\text{iterations} \end{array}$$

The data was assessed utilizing the FCM-DOA-SVM system to identify CLFP, comprising 504 images. The output of the algorithm training is shown in Table 7 (i.e., the test phase results of the proposed hybrid method are shown in this table). The FCM-DOA-SVM setup kept HOG cell-size at 32, HOG block-size at 2 and Dolphin population at 30 and it varied the Iteration threshold from 1 to 50.

**Table 7:** Outcome analysis of system testing with FCM-DOA-SVM.

**Exp. No**.	**Accuracy (%)**	**Processing time (s)**
1	96.0	1,388.09
2	96.26	1,369.73
3	96.0	1,370.13
4	96.0	4,614.75
5	96.0	1,323.98
6	96.0	1,344.68
7	96.0	1,357.67
8	96.0	1,387.29
9	96.0	1,369.75
10	96.0	1,323.13

The NCM-DOA-SVM model was trained on a dataset of 1,512 CLFP images, and its performance was assessed using standard evaluation metrics. The results are as given below:


$$\begin{array}{l} A_{CV}=95.1\pm 1.2\%,\;{\mathbb{R}}_{CV}=94.8\pm 1.4\%,\;S_{CV}=95.3\pm 1.1\%\\ \text{Bootstrap CI}=\left[93.9\%,96.3\%\right],\;\text{Convergence}=23\text{iterations} \end{array}$$

In order to analyze and identify the 504 images in the dataset as CLFP, the NCM-DOA-SVM system has been used. Table 8 lists the training phase results and provides an explanation of the testing phase results using the suggested hybrid approach. The following parameter settings were used when implementing the NCM-DOA-SVM system: The dolphin population level was kept at 30, the iteration threshold was investigated at 50, and the HOG cell-size was continuously set at 32 and the HOG block-size was fixed at 2.

**Table 8:** Outcome analysis of system testing with NCM-DOA-SVM.

**Exp. No**.	**Accuracy (%)**	**Processing time (s)**
1	98.0	1,341.57
2	98.0	1,351.05
3	98.0	1,348.48
4	98.0	1,339.74
5	98.0	1,357.74
6	98.0	1,345.81
7	98.0	1,348.91
8	98.0	1,318.66
9	98.0	1,389.36
10	98.0	1,378.92

The rating ratio rate is shown in Table 9 for the DOA method, as well as for the proposed hybrid approaches: DOA-SVM, FCM-DOA-SVM, and NCM-DOA-SVM.

**Table 9:** Comprehensive performance comparison of proposed methods.

**Method**	**Number of test images**	**Accuracy (%)**	**Processing time (s)**
DOA	504	91.00	3,902.533
DOA-SVM	504	94.07	5,039.768
FCM-DOA-SVM	504	96.03	1,684.920
NCM-DOA-SVM	504	98.00	1,352.024

### 4.2 Experimental analysis

The experimental results confirmed the robust performance of all proposed algorithms during the training phase. The best classification accuracy, 91.0%, was found for images with a size of 128 × 128 and a cell-size of 32 × 32, as shown in Table 5. Table 6 showed an improved classification accuracy of 94.07% for images of the same dimensions and cell-size when the number of dolphins matched the number of repetitions. Additionally, Tables 7, 8 reported a further increase in classification accuracy to 96.03 and 98.0% under the same conditions. These findings are corroborated by the data in Table 9. The DOA showed an average accuracy rate of 91.0%, whereas the DOA-SVM hybrid algorithm achieved an average accuracy of 94.07%, the FCM-DOA-SVM algorithm attained an average accuracy of 96.03% and NCM-DOA-SVM algorithm reached an average accuracy of 98.0%.

Our experimental findings indicate that the hybrid algorithm DOA-SVM demonstrates a higher classification ratio than the DOA. This improvement is attributed to the DOA optimization capabilities, which emulate the cooperative behavior of dolphins to enhance parameter selection. The algorithm improves convergence speed and reduces computational cost by learning from previous configurations. A well-tuned DOA balances exploration and exploitation, making parameter selection crucial for optimal performance.

According to experimental results, the suggested hybrid algorithm, which combines DOA-SVM with FCM, performs better in terms of classification accuracy than both the standalone DOA and the hybrid DOA-SVM algorithm. The efficient combination of FCM and DOA, where each technique improves the performance of the other, is the source of this improvement. When these algorithms are used together, they perform better on classification tasks and other tasks than when they are used separately. The FCM algorithm helps by providing soft aggregation capabilities, which enable more precise data point classification, particularly when features overlap. The DOA also supports more efficient searching, so that key data points can be identified and the required computations can be reduced.

Within the spectrum of assessed approaches, the NCM-DOA-SVM method consistently maintained superior classification accuracy. This enhanced performance is resulted due to the effective combination of NCM clustering, the DOA, and the SVM. This approach is convenient for handling unclear, overlapping and uncertain segments within the data and this quality is key for classifying complicated data sets. On the other hand, the DOA helps with global optimization, so features can be effectively chosen and parameters can be successfully tuned. Finally, the SVM provides good performance in the final classification stage. The integration of all three methods together creates a better and more efficient results than if only two are used, such as DOA or DOA-SVM.

**Figure 4:**
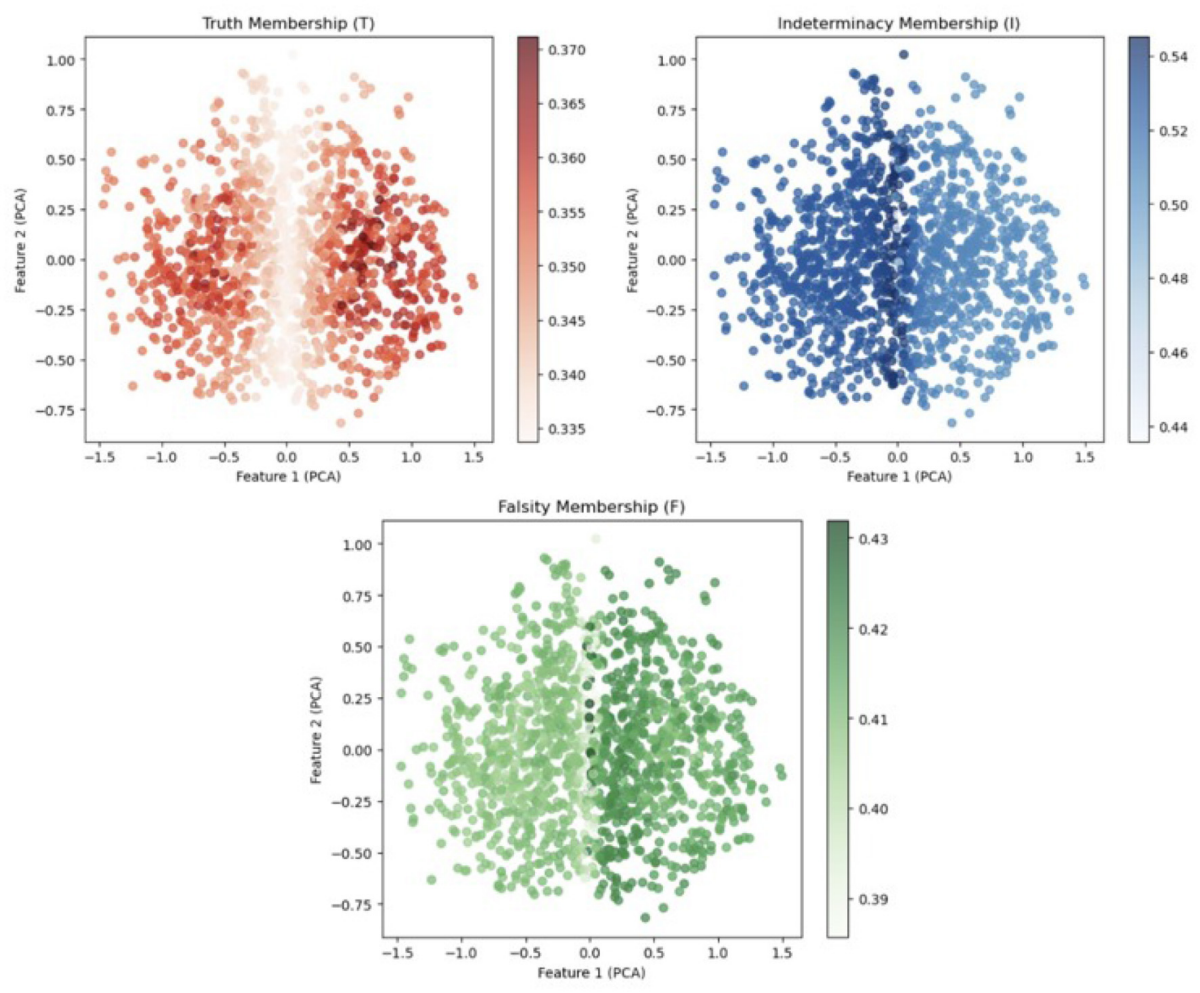
Neutrosophic logic T/I/F membership visualization.

In this neutrosophic logic visualization (see Figure 4), the T/I/F membership framework provides significant advantages for our database. Specifically, Truth membership (T) highlights robust and well-defined clusters at the core of the dataset, while Indeterminacy membership (I) captures transitional and boundary regions–effectively representing ambiguous cases that are often misclassified by traditional methods. Falsity membership (F) excels at identifying outliers, enhancing data integrity by revealing non-conforming patterns that may compromise analysis. The integrated T/I/F analysis enables a comprehensive and nuanced characterization of uncertainty, offering deeper insights and improved decision-making accuracy over conventional single-value approaches, thus establishing neutrosophic logic as a superior tool for extracting actionable information from uncertain databases.

In this work, we propose a CLFP recognition system based on the DOA with 3,902.533 processing seconds and 91.0% classification accuracy. Additionally, we combine the DOA and the SVM algorithms to propose a new hybrid algorithm. By suggesting a new fitness function based on the SVM algorithm that depends on dolphin values rather than the original parameters (G, ϵ, A), the combination aims to improve classification accuracy. This method took 5,039.768 s to process and had a classification accuracy of 94.07%. In order to further increase classification accuracy, we also suggest another hybrid algorithm that combines the DOA-SVM and the FCM algorithm. In comparison to both the DOA and the DOA-SVM algorithm, this algorithm efficiently handles ambiguous and imprecise data, resulting in a more accurate identification system and attaining classification accuracy of 96.03% with a processing time of 1,684.920 s. We suggest combining NCM with DOA-SVM in place of FCM. This results in a higher classification accuracy of 98.0% at a processing time of 1,352.024 s. The NCM-DOA-SVM outperforms other methods for CLFP images.

The comparative evaluation of bio-inspired optimization algorithms (see Table 10) reveals that DOA-SVM achieves the highest classification accuracy of 89.69% with the lowest standard deviation (±0.0132), demonstrating superior performance consistency compared to other metaheuristic approaches. While all bio-inspired methods substantially outperform the baseline SVM (88.33%), the computational cost varies significantly, with GA-SVM offering the best time-accuracy trade-off (89.26% in 1,547.74 ss) and PSO-SVM requiring the highest processing time (3,722.63 s) for comparable accuracy. The results validate DOA's effectiveness in hyperparameter optimization for CLFP recognition, though the 588-fold increase in processing time compared to baseline SVM highlights the computational overhead inherent in population-based optimization approaches.

**Table 10:** Performance comparison of bio-inspired optimization algorithms with SVM.

**Algorithm**	**Mean accuracy (%)**	**Standard deviation**	**Mean processing time (s)**
Baseline SVM	88.33	±0.0171	3.52
**DOA-SVM**	**89.69**	±0.0132	2073.12
PSO-SVM	89.65	±0.0136	3,722.63
GA-SVM	89.26	±0.0111	1,547.74
ACO-SVM	89.45	±0.0142	3,304.97

Bold values indicate the best (highest-performing) results among the compared methods.

## 5 Conclusion and future work

This study demonstrates that strategic hybridization of bio-inspired optimization with machine learning classifiers significantly advances contactless fingerprint recognition. Our systematic evaluation reveals clear performance progression: DOA (91.00%), DOA-SVM (94.07%), FCM-DOA-SVM (96.03%), and NCM-DOA-SVM (98.00%), with the NCM-based hybrid achieving superior accuracy while maintaining competitive processing efficiency.

This work makes several key contributions to the field of contactless biometrics. Primarily, it introduces a novel hybrid framework that successfully merges the Dolphin Optimization Algorithm with a Support Vector Machine classifier, establishing a new approach for CLFP recognition. The research is further advanced by integrating fuzzy clustering to refine feature representation and, most significantly, by pioneering the application of Neutrosophic C-Means clustering to effectively manage the inherent uncertainty and indeterminacy in fingerprint images. Beyond accuracy improvements, a thorough analysis of computational efficiency is provided, offering valuable practical insights that bridge the gap between algorithmic innovation and real-world system deployment.

Despite the promising results, this study has certain limitations. The generalizability of our findings is constrained by the dataset's limited size and demographic diversity, which may not fully represent broader populations. Furthermore, the robustness of the single-stage classification pipeline requires further validation against low-quality, noisy, or incomplete fingerprint images. To address these constraints and advance this research, future efforts should prioritize several key areas. Expanding the evaluation to include larger, multi-ethnic datasets is crucial for verifying performance across diverse real-world conditions. Exploring multi-stage architectures could also enhance robustness by decoupling processes like feature enhancement and classification. Finally, developing real-time prototypes would be invaluable for assessing the practical deployment of these methods, particularly under computational constraints. These research directions are essential for translating the current algorithmic innovations into reliable, real-world biometric systems.

## Data Availability

The original contributions presented in the study are included in the article/supplementary material, further inquiries can be directed to the corresponding author.
